# Elucidating reaction dynamics in a model of human brain energy metabolism

**DOI:** 10.1371/journal.pcbi.1013504

**Published:** 2025-09-24

**Authors:** Dimitrios G. Patsatzis, Efstathios-Al. Tingas, Subram Mani Sarathy, Dimitris A. Goussis, Renaud Blaise Jolivet

**Affiliations:** 1 Modelling Engineering Risk & Complexity (MERC), Scuola Superiore Meridionale (SSM), Napoli, Italy; 2 School of Computing, Engineering and the Built Environment, Edinburgh Napier University, Edinburgh, United Kingdom; 3 King Abdullah University of Science and Technology (KAUST), Clean Combustion Research Center (CCRC), Thuwal, Saudi Arabia; 4 Department of Mechanical Engineering, Khalifa University of Science, Technology and Research (KUSTAR), Abu Dhabi, United Arab Emirates; 5 Maastricht Centre for Systems Biology (MaCSBio), Maastricht University, Maastricht, The Netherlands; University of Edinburgh, UNITED KINGDOM OF GREAT BRITAIN AND NORTHERN IRELAND

## Abstract

Energy metabolism is essential to brain function and Bioinformatics, but its study is experimentally challenging. Similarly, biologically accurate computational models are too complex for simple investigations. Here, we analyse an experimentally-calibrated multiscale model of human brain energy metabolism using Computational Singular Perturbation. This approach leads to the novel identification of functional periods during and after synaptic activation, and highlights the central reactions and metabolites controlling the system’s behaviour within those periods. We identify a key role for both oxidative and glycolytic astrocytic metabolism in driving the brain’s metabolic circuitry. We also identify phosphocreatine as the main endogenous energy supply to brain cells, and propose revising our view of brain energy metabolism accordingly. Our approach highlights the importance of glial cells in brain metabolism, and introduces a systematic and unbiased methodology to study the dynamics of complex biochemical networks that can be scaled, in principle, to metabolic networks of any size and complexity.

## Author summary

The human brain is an energy-hungry organ, and understanding how it fuels its activity is essential for interpreting brain imaging techniques such as fMRI or PET. In this study, we combined a detailed mathematical model of brain metabolism with a powerful computational tool (Computational Singular Perturbation) that can disentangle the many overlapping processes occurring in brain cells. Our analysis revealed three main insights. First, very fast energy changes directly linked to neuronal activity occur within seconds, but these signals are too brief to be detected by most imaging methods. Instead, what brain scans often capture are slower processes in supporting cells (astrocytes) and the longer-lasting contribution of the creatine phosphate shuttle, which helps neurons restore their energy balance. Second, our results suggest that both glucose and lactate act as primary fuels for the brain, while phosphocreatine plays a central role as an immediate energy reserve. Altogether, our work calls for a revised view of brain energetics and provides a framework for better interpreting brain imaging signals.

## Introduction

The brain is an excitable organ with high energy metabolism, responsible for 20% of the body’s energy consumption, more during development [1]. Understanding brain energy metabolism is crucial because of its role in neurodegeneration [2], as an evolutionary [3,4] and behavioural [5–7] constraint, and because of its direct relation to computing [2,8–11].

Brain energy metabolism is underpinned by different cell classes with distinct roles. In particular, glial cells are responsible for metabolically supporting neurons [12–19]. In turn, such support has been linked to learning and behaviour [20–22].

Studying brain energy metabolism at the cellular level is rendered difficult by the experimental challenges of disentangling the role of different but interconnected cell types and reactions [23]. This has led to, on occasion, opposing views on the detailed organization of brain energy metabolism [15]. Similarly, while computational modeling can play a role, biologically accurate models are often relatively complex, reflecting biological complexity. Due to this intrinsic complexity, it has been difficult to extract meaningful biological information from such models [24–29].

Here, we address this problem using computational singular perturbation (CSP) to algorithmically identify the dominant dynamics of this model after synaptic activation in key functional periods, along with the metabolites and reactions that control the model’s behaviour during these periods. CSP is an algorithmic method designed to gain insight into complex multiscale networks of chemical reactions modeled as a set of ODEs [30,31]. CSP decomposes the dynamics of the system in multiple modes, classifying them as exhausted or active modes. The exhausted modes associate to the fastest time scales and to a number of equilibria that are generated in phase-space, while the active modes drive the system within the established equilibria and associate to the slower time scales, the faster of which characterize the evolution of the system. This classification of modes is meaningful when a wide range of timescales develops in the dynamics of complex chemical networks [32,33], such as those in the brain. CSP has been applied in a variety of other contexts [34–37].

Our analysis reveals the differing temporal dynamics of neuronal and glial energy metabolism, and the central role of glial oxidative and glycolytic metabolism in driving the observable behaviour of the system at longer timescales. Our analysis also reveals a central role for phosphocreatine as the main endogenous energy store for brain cells. These results illustrate the importance of investigating brain energy metabolism as a network of interconnected neurons and glial cells, of considering metabolic networks in their entirety beyond the metabolites that have been in the focus in the literature so far, and of considering the full dynamics of the system at multiple timescales.

## Results

**Fig 1:**
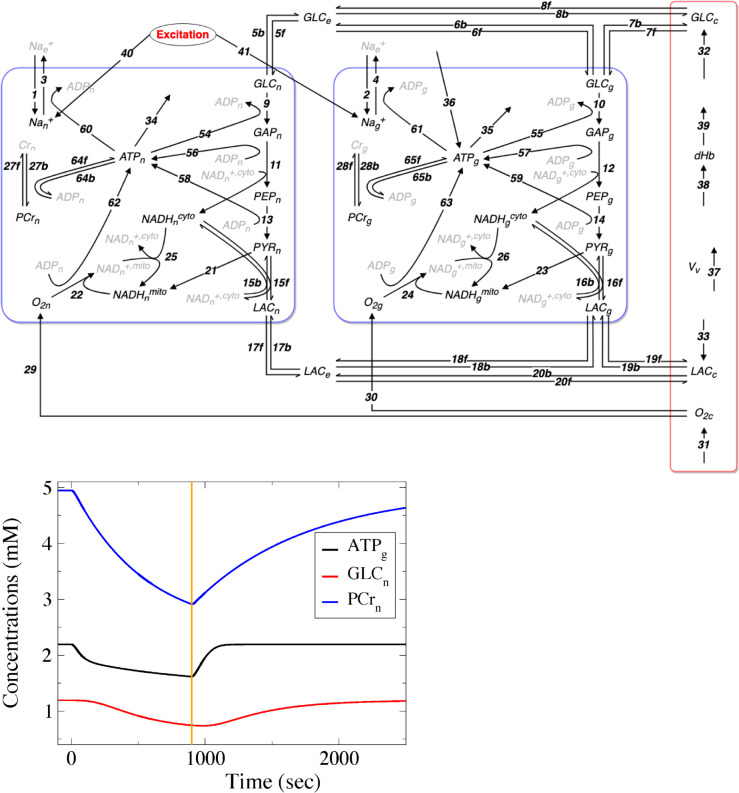
Schematic representation of the model and time course of the concentration for indicative metabolic species. **Top.** The model is divided in four main compartments: a neuronal compartment, an astrocytic compartment, the extracellular space and a vascular compartment. Neurons and astrocytes are further divided between a cytosolic sub-compartment and a mitochondrial sub-compartment to account for the compartmentalization of oxidative and glycolytic metabolisms. Both neurons and astrocytes contain a metabolic network including glycolytic enzymes, lactate dehydrogenase, glucose and lactate transporters, NADH shuttles, oxidative metabolism, phosphocreatine and the Na,K-ATPase electrogenic pump. The system as a whole is activated by two independent inputs. First, a glutamatergic presynaptic population activates AMPA receptors on the neuronal membrane and excitatory amino-acid transporters (EAATs) on the astrocytic membrane; modeled by reactions 40 and 41, respectively. The activation of both AMPA receptors and EAATs leads to an increase of the intracellular sodium concentration, which activates the energy consuming Na,K-ATPase pump and subsequent metabolic processes. Second, the cerebral blood flow (CBF) is modulated as a separate input, regulating the input/output flow of all metabolites in the vascular compartment; see Methods for further details. Finally, the extracellular space is the place where cells and capillaries exchange metabolites (metabolites written in gray are not variables of the model, black ones are). **Bottom.** Concentration profiles of indicative metabolites, demonstrating the wide range of timescales characterizing different periods of the process.

The biophysical model used to simulate the energy metabolism of the human brain [25] consists of four main compartments (neurons, astrocytes, extracellular space, and vasculature), as schematically illustrated in Fig 1 and detailed in the Methods section. That model is, to the best of our knowledge, one of the most detailed biophysical models of brain energy metabolism. It has been extensively validated against *in vivo* experiments (see Fig A in S1 Text), and currently is the foundation for designing larger and more complex models [24].

### Computational singular perturbation analysis

The model considered here can be simulated in a number of scenarios. Here, we focus exclusively on an *in vivo* human activation scenario. Briefly, we assume a situation similar to a typical experimental protocol, with a subject initially at rest, before being stimulated for an extended period of time, and finally returning to rest [38]. Our simulations thus contain two main epochs of interest: the *activation epoch* (lasting 900 s), when external input to the cortex drives first neuronal activity and then brain energy metabolism, and the *post-activation epoch* (from 900 sec onward), after external input to the cortex is over, during which brain energy metabolism is still recovering owing to its naturally slow dynamics. The behaviour of indicative variables of the model in these two epochs can be seen in Fig 1, where the multiscale character of the process is demonstrated.

**Fig 2:**
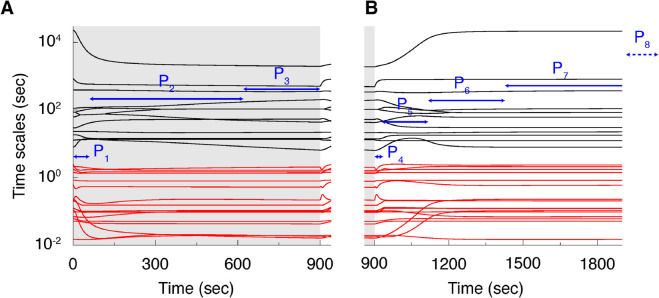
Fast and slow timescales of the model. The timescales of the model during the activation epoch (**A**; *t* = 0−900 s) and post-activation epoch (**B**; *t*>900 s). CSP automatically defines periods of interest *P* (blue arrows; see text), in which specific modes drive the process. In each period *P*_1_-*P*_8_, the timescales that stand directly below the blue arrows correspond to fast, exhausted modes, while the slow timescales stand above the blue arrows. The very late stage of the post-activation epoch (period P_8_; *t*>5730 s) is not shown here. Modes for which the timescale is fast in all periods and epochs are drawn in red.

We now apply the CSP algorithm to these *in silico* results (see Methods). The first output of CSP is the identification of the characteristic timescales of the system (Fig 2). CSP algorithmically defines distinct periods in which the number of exhausted fast modes can be considered constant, as denoted by blue arrows in Fig 2. During a specific period, the timescales in the dynamics of the model that sit below the blue arrows in Fig 2 relate to the exhausted modes that characterize the development of equilibria. Note that in the context of the current work, an *equilibrium* refers to a mapping among all reaction rates in which significant cancellations are encountered among additive terms. For instance, a typical example is the *partial equilibrium* between the two directions of a reversible reaction [39,40]. These equilibria define a low dimensional surface in phase-space (known as manifold M), on which the slow evolution of the system is confined. On the other hand, the timescales standing above the blue arrows in Fig 2 characterize the action of the active (slow) modes that govern the system’s evolution on this manifold. In each period, the characteristic timescale is related to the dominant of the active modes; i.e. the mode that has the largest impact in driving the process (see Methods for the definition of exhausted, characteristic and active modes).

When applied to the model in Fig 1, this procedure yields three separate periods *P*_1_ to *P*_3_ in the activation epoch (*P*_1_: 0-60 s; *P*_2_: 60-620 s; *P*_3_: 620-900 s), and five periods in the post-activation epoch (*P*_4_: 900-935 s; *P*_5_: 935-1123 s; *P*_6_: 1123-1433 s; *P*_7_: 1433-5730 s; *P*_8_: beyond 5730 s). As one would expect, the number *M* of exhausted modes increases monotonically within each epoch (*M*_*P*1_=17, *M*_*P*2_=26 and *M*_*P*3_=27 in the activation epoch; *M*_*P*4_=17, *M*_*P*5_=22, *M*_*P*6_=26, *M*_*P*7_=27 and *M*_*P*8_=28 in the post-activation epoch), indicating that during the course of each epoch, the system is progressively driven towards a full equilibrium. At the transition between the activation and post-activation epochs, however, the number of exhausted modes decreases, due to the perturbation generated by the sudden interruption of the external stimulation. Identification of these periods and exhausted modes *M* along with the associated equilibria is performed locally along the dynamic trajectory that originates from the chosen initial conditions. While the analysis at any given moment depends on the system’s current state, the entire trajectory — and thus the sequence of equilibria — is contingent on the starting point. The results presented here are therefore specific to the dynamics evolving from the physiologically relevant resting state chosen for this study.

In each of these eight periods, it is now possible to identify (i) the reactions that participate in the established equilibria and the metabolic species associated to them (i.e., the species that respond the fastest when these equilibria are perturbed), and (ii) the reactions and metabolic species that relate to the active modes that drive the system.

#### Behaviour of the model in the activation epoch.

**Fig 3:**
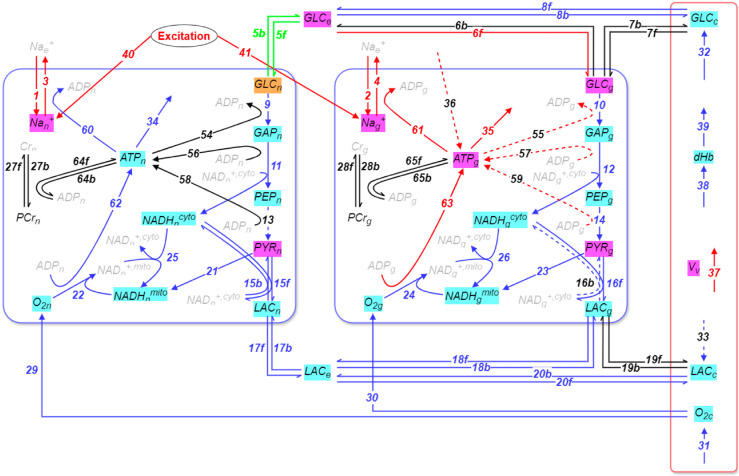
Equilibria among reactions and fast metabolic species in periods $P_{1}$ to $P_{3}$ (activation epoch). In each period, a number of equilibria are formed between various reactions. When subjected to a perturbation, the response of these equilibria manifests in significant adjustments in the concentration of a number of metabolic species. These reactions and species are identified by the *API* and *Po* tools, respectively (*Amplitude Participation Index* and *Pointer*; see Methods). Shown here are reactions exhibiting 2%≤|API|≤9% (dashed lines) and |API|>9% (solid lines). The metabolic species highlighted are those exhibiting Po>45%. These reactions and metabolites are additionally color-coded in three subgroups, blue / cyan for the 17 equilibria established in *P*_1_, red / magenta for the additional 9 equilibria established in *P*_2_, and green / orange for the last equilibria established in *P*_3_. Note that the presynaptic excitatory activity (reactions 40 and 41), contributes to the equilibrium formed in period *P*_2_, but not to the equilibria formed in period *P*_1_, indicating that this external drive to the system does not contribute in driving the system after *P*_2_. See also Fig B in S1 Text.

We first turn our attention to the activation epoch. Fig 3 and Table 1 display the key results for the three periods in this epoch. Fig 3 displays the fast species, around which equilibria are established, and the reactions participating in these equilibria, while Table 1 details the species and reactions related to the mode dominating the slow dynamics during each of the three periods of the activation epoch. We focus first on the equilibria.

**Table 1:** Species and reactions indicated by the CSP diagnostic tools (*Po* and *TPI*) to contribute to the dominant active mode during the three periods *P*_1_ to *P*_3_ in the activation epoch (see Fig 1).

${𝐏}_{1}$ period 18th mode				${𝐏}_{2}$ period 27th mode				${𝐏}_{3}$ period 28th mode			
Species	*Po*	React.	*TPI*	Species	*Po*	React.	*TPI*	Species	*Po*	React.	*TPI*
${ATP}_{g}$	0.869	63	20.0 %	${GLC}_{n}$	0.783	5b	–16.4 %	${PCr}_{n}$	0.999	27f	–48.4 %
		61	–19.2 %	${GLC}_{g}$	0.072	63	16.4 %			27b	–21.3 %
		35	–18.3 %	${GLC}_{e}$	0.061	61	–13.2 %			64f	8.6 %
		10	–14.3 %			35	–12.6 %			62	–5.0 %
		36	6.7 %			8b	–8.9 %			60	–4.1 %
		12	6.7 %			5f	6.1 %			64b	3.7 %
		14	5.8 %			59	4.7 %			34	–3.1 %
						57	4.7 %				
						55	–4.7 %				
						36	4.6 %				

The indicators *Po* and *TPI* have been calculated at representative time points for each period (t=25 s for *P*_1_, t=500 s for *P*_2_ and t=850 s for *P*_3_). Contributions smaller than 3% are neglected.

Fig 3 shows that about two thirds of the equilibria in the activation epoch are established within 60 s in *P*_1_ (*M*_*P*1_=17), another third is added in *P*_2_ (*M*_*P*2_ − *M*_*P*1_=9), and one last one in *P*_3_. Major participants in the equilibria established in *P*_1_ are many reactions in the neuronal and astrocytic compartments, and in the extracellular space. This points to two conclusions. First, it suggests that persistent presynaptic activation to a brain region tends to rapidly drive the local metabolic network from its baseline steady state to an alternate steady state. This is consistent with observations of metabolic variables reaching plateaus upon long stimulation episodes [41], and with the presence of various adaptation and desensitization mechanisms at synapses and in neurons [42]. Second, it suggests that this transition is largely complete within the initial period *P*_1_. Of note, most of the fast modes in *P*_1_ have a related timescale below 1 s, with only 4 of them having a timescale slightly above 1 s but below 2 s (Fig 2). In the Discussion, we argue that this is a crucial fact for the interpretation of functional brain imaging data. In particular, the reactions involving O_2_ consumption and transport (from the vasculature to the parenchyma and cells; reactions 22, 24 and 29 to 31) exhibit a significant participation in the equilibria that are established in *P*_1_ and this lasts up to *P*_3_, while – as it will be shown next - they do not significantly contribute in driving the system. Similarly, transport of lactate from the vasculature to cells through parenchyma (reactions 17-18 and 20), as well as lactate to pyruvate conversion in both astrocytes and neurons (or vice-versa; reactions 15 and 16), all exhibit a similar behaviour. Additionally, reactions comprising the glycolysis in both the neuronal and glial compartments (reactions 9-14), as well as reactions involved into the energetic metabolism of NADH in astrocytes and neurons (reactions 21-26) and ATP in neurons (reactions 60, 62, 34), participate significantly in the equilibria that are formed early in *P*_1_.

By contrast, in *P*_2_ the additional equilibria include the Na,K-ATPase (reactions 3-4), which is responsible for a large fraction of the brain’s energy budget and is the main driver of metabolic activation following presynaptic activity [2,43–45]. This is however expected, owing to the relatively slow time constant for sodium extraction (in the tens of seconds). In addition, sodium leakage through the membrane (reactions 1 and 2) and the presynaptic activation (reactions 40–41) also contribute to the established equilibria along with the energetic metabolism of ATP in astrocytes (reactions 61, 63, 35). Finally, in *P*_3_, a last equilibrium is formed in the network, which interestingly involves the export of glucose from the neuronal compartment to the extracellular space and back (reaction 5).

Despite similarities in established equilibria between the neuronal and glial compartments, there are some notable differences. These include equilibria involving ATP being established first in neurons in *P*_1_ (reactions 60, 62, 34), and then later in astrocytes in *P*_2_ (reactions 61, 63, 35). While shuttling of lactate through the extracellular space is established fast during *P*_1_, this is not the case for the shuttling of glucose. In fact, Fig 3 shows that this pathway is only very gradually established as an equilibrium (reaction 8 in *P*_1_, 6f in *P*_2_ and 5 in *P*_3_). These observations indicate that lactate is a faster fuel for neurons than glucose, as already suggested by others [46]. Because ADP is a hub molecule, reactions involving ADP do not, or only weakly, participate in equilibria (reactions 54-59 and 64-65).

We now turn to the slow dynamics of the system. Table 1 presents, for each period *P*_1_ to *P*_3_, the reactions contributing the most to the dynamics of the dominant active mode. This is the mode that most strongly influences the evolution of the system within the established equilibria. The related timescale is now the characteristic timescale of the system. These driving reactions were identified by the *Timescale Participation Index* (*TPI*; see Methods), which assesses the percentage contribution of each reaction to the amplitude of the characteristic timescale. This index is scaled, so that the net sum of the absolute value of all *TPI*s equals a hundred, and its sign denotes whether the contribution of the related reaction leads towards (negative) or away (positive) from homeostasis. A mode is declared dissipative or homeostatic when the sum of all *TPI*s is negative, and explosive when that sum is positive (see Eq (8) in Methods). The timescales characterizing the modes displayed in Table 1 are all dissipative in nature, relating to modes that will all eventually become exhausted. Table 1 also reports the metabolic species mostly related to the characteristic mode. This is captured by the *CSP Pointer* (*P*_0_), which is scaled so that the sum of all pointers equals unity (see Eq (9)). These are the species mainly involved in the reactions driving the process via the characteristic mode.

Already in *P*_1_, the main reactions and species identified by CSP point to the astrocytic Na,K-ATPase and oxidative energy metabolism as the key drivers for the behaviour of the system, with *ATP*_*g*_ being the main metabolite associated with this mode. The main contributions come from either the production (63; astrocytic oxidative metabolism), or consumption (35 and 61; astrocytic Na,K-ATPase), of astrocytic ATP (see Tables 4 and 5). Consumption of astrocytic ATP by the Na,K-ATPase is indirectly driven by the uptake of glutamate, and thus reflects the amplitude of the presynaptic input to the local circuitry, but not postsynaptic computation. The homeostatic character of that mode is mainly promoted by reactions that consume *ATP*_*g*_ (61 and 35 and 10), and is opposed by those that produce it (63 with minor contributions from reactions 36, 12 and 14). From the above, we observe that some of the reactions with significant *TPI* values related to the characteristic mode’s timescale were also previously reported contributing to the equilibria established in that period (see Fig 3). This suggests that a reaction (in this case 10, 12 and 14) can contribute both to the fast and slow dynamics in a given period. In other words, these reactions contribute to the formed equilibria in the fast subspace, yet their projection to the slow subspace is non-negligible, thereby also affecting the slow evolution of the system.

The characteristic slow mode in *P*_2_ shares similarities to the one in period *P*_1_. In *P*_2_, the characteristic mode is associated principally to neuronal glucose (*GLC*_*n*_), with contributions from some of the same reactions involved in *P*_1_ (63, 61, 35) related to the production, or consumption, of astrocytic ATP, and with a comparable contribution from neuronal glucose uptake (5b). Here, the dissipative character of the timescale is promoted by reactions that consume *ATP*_*g*_ (61, 35, 55), and opposed by reactions that produce *ATP*_*g*_ (63, 59, 57, 36). However, these contributions cancel out. The dissipative character of the mode is thus promoted by the *GLC*_*n*_-consuming reaction 5b and the *GLC*_*e*_-consuming reaction 8b, and is opposed by the *GLC*_*n*_-producing reaction 5f. Note that similar to *P*_1_, some of the reactions with large *TPI* values were also identified in the equilibria formed in the same period (e.g., 8b, 35, 61, 63, etc...).

Finally, in *P*_3_, the characteristic slow mode is almost exclusively related to the neuronal phosphocreatine *PCr*_*n*_. Here, the dissipative character of the timescale is mainly due to the forward and backward directions of the creatine-phosphocreatine shuttle (reaction 27).

As noted above, the sign of *TPI* in Table 1 indicates whether a process drives the system towards an equilibrium (negative *TPI*), or away from one (positive *TPI*). The distribution of signs in Table 1 indicates the presence of competing processes driving the system towards a new equilibrium (reactions 10, 35 and 61 in *P*_1_, and 5b, 8b, 35 and 61 in *P*_2_), or away from it, as reaction 63, which models production of ATP by glial oxidative metabolism, does. This reinforces our interpretation that the slow behaviour of the system during activation is driven first by glial oxidative metabolism (it is slowed by the consumption of *ATP*_*g*_ in *P*_1_ and *P*_2_), then by neuronal glucose metabolism (the generation of *GLC*_*n*_ and *GLC*_*e*_ slows down this process), and finally by reaction 27.

In short, it appears that, upon sustained activation, brain energy metabolism rapidly transits from its baseline steady state to an ‘activated’ state, with reactions typically associated to postsynaptic neuronal activities by proxy, such as oxygen transport and neuronal oxidative metabolism, equilibrating within seconds and playing no significant role in the subsequently observable slow behaviour of the system. Instead, we observe that the activation epoch divides in 3 periods, with glial oxidative metabolism partially dominating the slow dynamics of the system in the first two periods (in particular reactions 35, 61, 63), and the neuronal creatine phosphate shuttle dominating it in the third and last period of that epoch. These results, taken together with the fact that all the fast modes equilibrating in period *P*_1_ do so with timescales of a few seconds or less, suggest that experimental methods that rely on *in vivo* brain energy metabolism with sampling frequencies of approximately 1 Hz or slower [47] are in fact incapable of capturing the fast transitions in energy metabolism directly associated to neuronal activity. Instead, they probably mostly capture secondary glial energy metabolism, and the neuronal creatine phosphate shuttle in late stages of activation (after more than 10 min of activation here). Additionally, this suggests that the neuronal creatine phosphate shuttle captures the long-tail of post-activation recovery and is directly related to the metabolic cost incurred by activated neurons, while the fact that glial oxidative metabolism appears to dominate the early activation period is directly related to presynaptic activity via the glutamate – glutamine cycle.

Next, we turn to the post-activation epoch.

#### Behaviour of the model in the post-activation epoch.

**Fig 4:**
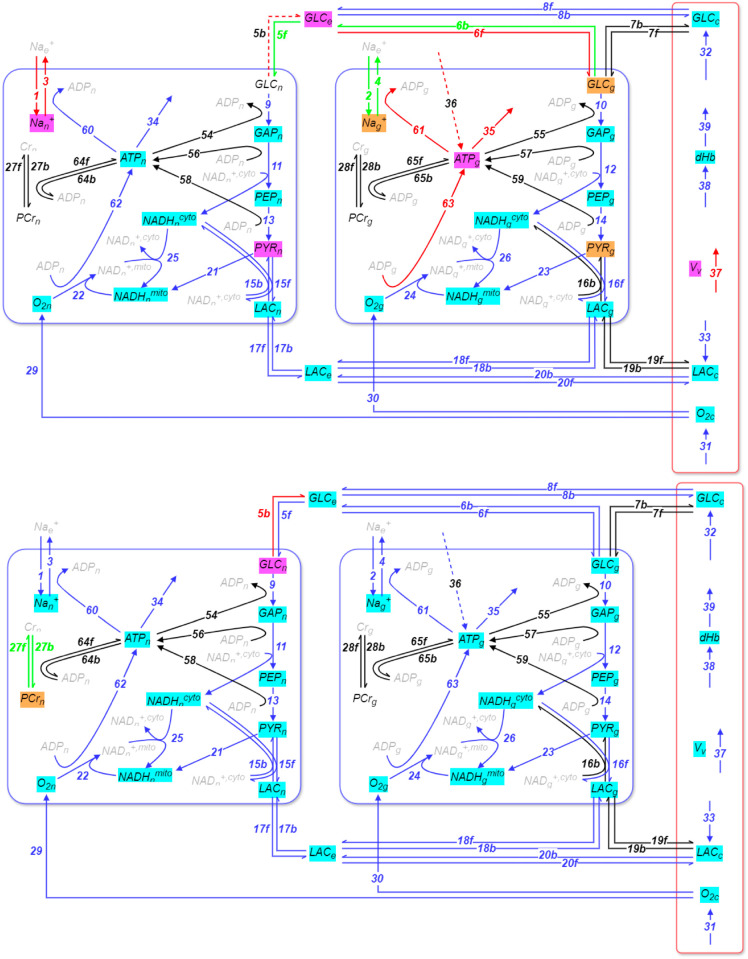
Equilibrating reactions and fast metabolic species in the post-activation epoch periods 4 to 6 (top) and 7 to 8 (bottom). In each period, a number of equilibria are formed between various reactions, which relate to specific metabolic species. These reactions and species are respectively identified by the *API* and *Po* tools (see Methods). The reactions shown exhibit |API|>9% (solid lines) and 2%≤|API|≤9% (dashed lines), while related species are those exhibiting Po>45% (Top: Blue / Red / Green reactions and Cyan / Magenta / Orange-highlighted species refer to the equilibria established in *P*_4_/*P*_5_/*P*_6_, respectively. BOTTOM: Red/Green reactions and Magenta/Orange-highlighted species refer to the 1/1 equilibria established in *P*_7_/*P*_8_, respectively. See also Figs C and D in S1 Text.

Fig 4, and Tables 2 and 3 display the key results for periods *P*_4_ to *P*_8_ in the post-activation epoch (Fig 4 refers to periods *P*_4_-*P*_6_ (top) and *P*_7_-*P*_8_ (bottom)), again highlighting the reactions and metabolic species that contribute to the emerging equilibria. Tables 2 and 3 present the steps that contribute the most to the mode that drives the evolution of the system in each of the periods *P*_4_ to *P*_8_.

**Table 2:** Output of the CSP diagnostic tool for the dominating slow mode during periods *P*_4_ to *P*_6_.

${𝐏}_{4}$ period				${𝐏}_{5}$ period				${𝐏}_{6}$ period			
Species	CSP *Po*	React.	*TPI*	Species	CSP *Po*	React.	*TPI*	Species	CSP *Po*	React.	*TPI*
${ATP}_{g}$	0.548	10	–28.5 %	${Na}_{g}^{+}$	0.881	4	–38.6 %	${GLC}_{n}$	0.834	5b	–42.3 %
${GLC}_{g}$	0.422	12	10.3 %	${GLC}_{g}$	0.050	10	–8.9 %	${GLC}_{g}$	0.093	8b	–23.4 %
		14	10.3 %	${PYR}_{g}$	0.042	2	–8.8 %	${GLC}_{e}$	0.072	5f	16.2 %
		59	–8.3 %			63	7.6 %			9	–4.1 %
		57	–8.3 %			35	–7.5 %			6b	–3.7 %
		55	8.3 %							6f	3.2 %
		61	–7.2 %								
		63	7.0 %								
		35	–5.7 %								

The indicators CSP *Po* and *TPI* have been calculated at representative time points for each period (t=950 s for *P*_4_, t=1000 s for *P*_5_ and t=1300 s for *P*_6_). Smaller contributions are neglected. Periods *P*_4_ to *P*_8_ divide the post-activation epoch in *P*_4_: 900−935 s, *P*_5_: 935−1123 s, *P*_6_: 1123−1433 s, *P*_7_: 1433−5730 s and *P*_8_: 5730 s – end of the process.

**Table 3:** Output of the CSP diagnostic tool for the dominating slow mode during periods *P*_7_ and *P*_8_.

P_7_ period				P_8_ period			
Species	CSP *Po*	React.	TPI	Species	CSP *Po*	React.	TPI
PCr _*n*_	0.991	27 b	−46.4%	PCr _*g*_	0.99	28 f	−48.0%
		27 f	−43.4%		28 b	−47.4%	
		62	−4.0%				

The indicators CSP *Po* and *TPI* have been calculated at representative time points for each period (t=2000 s for *P*_7_ and t=6500 s for *P*_8_). Smaller contributions are neglected.

The post-activation epoch starts with the relatively short, 35 s-long, period *P*_4_. Fig 4 shows that the fast exhausted modes in *P*_1_ (red) all remain in that category in *P*_4_, with very similar associated timescales as in *P*_1_, i.e. mostly below 1 s, except for the same 4 modes as in *P*_1_ with timescales between 1 and 2 s. During the next two post-activation periods, *P*_5_ and *P*_6_, which last ∼500 s, the fast exhausted modes introduce the same number of equilibria as in the activation period *P*_2_. Another equilibrium is added in the long-lasting *P*_7_, similarly to the one added in *P*_3_, while a final equilibrium is established in *P*_8_, which shares no similarities with the activation epoch. A comparison between Figs 3 and 4 reveals very similar equilibria in the metabolic network between the activation and post-activation periods, P_1_ and P_4_, *P*_2_ and *P*_6_, and *P*_3_ and *P*_7_, respectively. In the following, we only highlight the most significant differences between the two epochs.

A comparison between *P*_1_ and *P*_4_ reveals that the metabolic species involved in the formation of the equilibria are the same. The same applies for the established equilibria, which are formed mainly between the reactions related to O_2_ consumption and transport, lactate transport, the glycolysis, and the energetic metabolism of NADH in both neurons and astrocytes. A notable exception, is the conversion of lactate to pyruvate in astrocytes, which does not contribute to the equilibria in *P*_4_, while it does in *P*_1_.

Next, comparing *P*_2_ and *P*_6_, we observe again a similar behaviour. The newly established equilibria relate to the pathways involved in glucose transport between compartments, the Na,K-ATPase pump, and ATP production in astrocytes. Notably, it is shown that the equilibria related to the Na,K-ATPase pump (reactions 1-4 in Fig 4) in the post-activation epoch are adjusted to the absence of excitation characterizing this epoch (reactions 1-4 and 40-41 in Fig 3). These results suggest that the metabolic network adjusts itself back to its initial pre-activation metabolic equilibrium.

However, comparing periods *P*_3_ and *P*_7_, we observe a crucial difference in glucose transport. Although the equilibria are formed gradually in both epochs (reactions 8 in *P*_1_ and *P*_4_, 6 in *P*_2_ and *P*_5_, and 5 in *P*_3_ and *P*_7_), the transport of glucose from astrocytes to the extracellular space (reaction 6b) does not contribute to the equilibria during activation, but strongly does during post-activation. In addition, the transport of glucose from the extracellular space to neurons (reaction 5f) and to a lesser extent in the opposite direction (reaction 5b) contribute to the equilibria early on in *P*_6_ (see Fig 4), while they do so only during the final activation period *P*_3_ (see Fig 3). This finding suggests that the role of glucose as a direct fuel for both astrocytes and neurons is more significant in the post-activation epoch than in the activation epoch. This is also apparent in Fig A in S1 Text, where ${\text{CMR}}_{\text{glc}}$ decays back to its baseline much slower in that epoch than ${\text{CMRO}}_{2}$ (panels C and D).

Finally, in *P*_8_, which is only attained very late in the post-activation epoch, the equilibrium between reactions producing and consuming the phosphocreatine in neurons (reaction 27) is established.

We now turn to Tables 2 and 3 to investigate the reactions contributing mostly to the system’s characteristic timescale in the post-activation epoch. Table 2 shows that in periods *P*_4_ and *P*_5_ that immediately follow the activation epoch, the dynamics of the system is almost entirely dominated by the astrocytic metabolism, with the key metabolites associated to the fastest of the slow modes being *ATP*_*g*_ and *GLC*_*g*_ in *P*_4_, and *Na*_*g*_ in *P*_5_.

In *P*_4_, the system’s characteristic mode is mostly associated to the astrocytic glycolysis (reactions 10, 12, 14, 55, 57 and 59 (see Tables 2, 5 and 6), with a smaller contribution from astrocytic oxidative metabolism and the astrocytic Na,K-ATPase (35, 61 and 63; see Tables 2 and 6). In *P*_5_, that picture is very much conserved, with the dominating reactions being associated to the astrocytic metabolism, either glycolytic (reaction 10) or oxidative (reaction 63), with a significant contribution from the Na,K-ATPase (reactions 2, 3 and 35). These numbers suggest that, very much like in *P*_1_ and *P*_2_ earlier, whatever transitions occurring in neuronal energy metabolism in response to the discontinuation of the presynaptic stimulation and increased cerebral blood flow, are over within a few seconds and do not significantly contribute to the slow dynamics of the system. Instead, these numbers suggest that in both *P*_4_ and *P*_5_, the main driver of the fastest slow mode is the consumption of astrocytic ATP by the Na,K-ATPase, again indirectly driven by the uptake of glutamate and reflecting the amplitude of the presynaptic input to the local neuronal circuit, but not postsynaptic computation. The main difference between the activation and post-activation epochs, in that regard, is that the lead contribution comes from astrocytic oxidative metabolism in the activation epoch, while it comes from the astrocytic glycolytic metabolism is the post-activation epoch. Again, the distribution of signs in Table 2 indicates the presence of a competition between processes driving the system towards a new equilibrium, and those driving the system away from it. In *P*_4_, in particular, we see that reactions 12 and 14, both reactions part of the astrocytic glycolysis, provide the main opposing contributors to the driving of the system towards its equilibrium. We hypothesize that this is due to the availability of rapidly equilibrating oxygen in the activation epoch, and to the relative lack of it in the post-activation epoch. In the Discussion, we argue that this might be an important driver for the use of astrocytic glycogen post-activation.

In *P*_6_, the key metabolite associated to the system’s characteristic modes is glucose, mostly in the neuronal compartment (see Table 2) with major contributions from glucose transport across the neuronal and astrocytic membranes (see Table 5). This reflects continuously equilibrating glucose concentrations in different compartments of the parenchyma, while glucose uptake by the tissue is still higher than that at baseline (see Fig A (panel C) in S1 Text).

Finally, periods *P*_7_ and *P*_8_ show a very similar picture, but in two different compartments, and recapitulate partially the observations we made for the end of the activation epoch (period *P*_3_). Indeed, periods *P*_7_ and *P*_8_ are dominated by the creatine phosphate shuttle, first in the neuronal compartment (*P*_7_), then in the neuronal compartment (*P*_8_; see Table 3).

In summary, from a functional point of view, the post-activation epoch, even though it is composed of five distinct algorithmically-determined periods (*P*_4_ to *P*_8_), can be divided up in roughly 3 functional tranches. First (*P*_4_ and *P*_5_), the dynamics of the system is driven by astrocytic metabolism with a significant contribution of glycolytic metabolism, mostly reflecting the energy consumption due to glutamate uptake by the astrocytic compartment. Second, a transition tranche (*P*_6_) towards the establishment of equilibrated glucose concentrations in different compartments. Finally, in the last tranche (*P*_7_ and *P*_8_), the dynamics of the system is driven by the creatine phosphate shuttle, first in the neuronal compartment (*P*_7_), then in the astrocytic compartment (*P*_8_).

## Discussion

Brain energy metabolism is inherently complex, with different intricately connected cell types using similar intracellular metabolic machinery, and a wide spectrum of timescales. This organisation makes it difficult to study brain energy metabolism experimentally, or by using biologically accurate computational models. Here, we have demonstrated the power of combining such complex and multiscale models of metabolic circuits with automated algorithmic decomposition by Computational Singular Perturbation (CSP). CSP revealed several important key findings. First, the rapid (within seconds) equilibrating of reactions associated with neuronal postsynaptic activity. Second, in response to presynaptic input specifically, a key role for glial oxidative and glycolytic metabolism in driving the observable behaviour of the system following the onset or offset of activation. And third, a central role for phosphocreatine as an important energy store for brain cells.

These findings are consistent with the finding that the BOLD signal observed in fMRI is correlated to the input of an activated area rather than with local neuronal activity [48]. Our results are also consistent with experimental findings in positron emission tomography (PET), that metabolic responses upon activation, in particular tissue glucose uptake, are driven by astroglial glutamate transport [49]. Our results, taken with these experimental findings, highlight the need for a reevaluation of the role of astrocytes in brain imaging signals like BOLD fMRI and [^18^F]FDG PET. Far from the common interpretation that these signals measure local neuronal activity, the results mentioned above, suggest that they instead measure the amplitude of the synaptic input to an activated region and that they are mostly glial in nature. Our results additionally highlight the brief time window for detection of metabolic correlates of local neuronal activity at the onset of activation (Figs 2, 3 and Table 1). We find that in order to detect such signals, imaging methods need a sampling rate faster than ∼1 Hz.

Lastly, we identify an important role for the creatine phosphate shuttle in supporting brain energy metabolism, both in neurons and glial cells. These results prompt a reevaluation of the importance of phosphocreatine in brain energy metabolism. Interestingly, it is linked to a number of pathologies in other tissues with high-energy requirements [50], and to neurological [51] and developmental disorders [52]. In fact, our results should prompt a reevaluation of our view of brain energetics as a whole. The consensus view is that the brain is fuelled mostly by glucose, and contains a single fuel storage in the form of astrocytic glycogen. Our results suggest that instead, both glucose and lactate should be considered as brain fuels delivered via the bloodstream, consistent with experiments showing that blood lactate is an important fuel while exercising [53], when dealing with complex cognitive tasks [54], and a primary source of brain energy [55–59]. Second, while glycogen is degraded in astrocytes following stimulation, and used to support neuronal metabolism in the exercising brain [19], it has also been described as necessary to support long-term memory formation [22,60]. It is thus unclear how much of an energy-production support role it plays in normal physiology. In contrast, our results show that phosphocreatine, which rests at a relatively elevated concentration in brain tissue (∼5 mM), can rapidly be mobilised to restore ATP levels in both neurons and astrocytes, and might constitute a more immediate fuel storage than glycogen. We therefore propose that brain energy metabolism be revised to include both glucose and lactate as primary fuels, and phosphocreatine and glycogen as fuel stores. Furthermore, Swanson has suggested that both glycogen and phosphocreatine support the energetic yield of ATP during high energy demand [61], reinforcing the view that brain energy metabolism can only be understood when considering also their dynamics and that of related metabolites (such as inorganic phosphate and ATP).

Our results illustrate the power of CSP to extract biologically meaningful information from complex computational models. While it still requires interpretation of the generated diagnostics by a field specialist, it provides an unbiased feature set to do so. CSP is not limited to metabolism or neurobiology, and can be scaled to more complex models easily.

## Methods

### Mathematical model of brain energy metabolism

We simulated the mathematical model of human brain energy metabolism by Jolivet et al. [25] (see Fig 1). The model consists of a neuronal compartment, an astrocytic compartment, an extracellular space compartment, and a vascular compartment. The original model by Jolivet and colleagues can be simulated in a number of different scenarios, but here we focus exclusively on the scenario corresponding to *in vivo* human brain activation. In order to analyze the model with the CSP tools, we translated the original implementation from Matlab (available at https://github.com/JolivetLab) to Fortran, and ensured that both codes produced the same solutions (see Fig A in S1 Text). We refer the interested reader to Ref [25] for a full detailed description of the model, as well as for a detailed explanation of how the model was calibrated and validated against experimental data. The Fortran implementation (available at https://github.com/patsatzisdim/humBEMm_CSP) was coupled to standard BLAS/LAPACK routines to ensure seamless integration with our CSP analysis pipeline. The CSP methodology and diagnostics (see below) are language-agnostic and operate solely on the model’s ODE system and Jacobian. In the following, we only very briefly describe the human simulation case.

The model is formulated as a system of 29 differential equations (see Table 4), describing the evolution of the metabolic species within the four compartments of the model; the subscripts *n*, *g*, *e* and *c* denote the compartment - neuronal, glial (astrocytic), extracellular and vascular (capillary) - where each metabolic species sits. Note that the equations for the neuronal membrane voltage, ion channel gating variables, and neuronal intracellular calcium variables included in [25] are not considered in the case of human brain activation. The variables, their steady-state values and the corresponding governing equations (written as a stoichiometric sum of reaction rates, see Eq (4)) are given in Table 4. The model includes a total of 65 reactions, which are either unidirectional or appear as a forward-backward pair. The expressions of the respective reaction and transport rates *R^k^* for k=1,…,65 are provided in Tables 5 and 6. Table 5 includes all the reaction and transport rates appearing in the governing equation of all but the adenosine triphosphate (ATP), while Tables 6 includes the reactions producing or consuming ATP. This distinction is made because in Ref [25], the stoichiometry of the fluxes governing the evolution of ATP is dependent on certain variables of the model. As shown in the rightmost column of Tables 6, all fluxes in [25] include the multiplying factors $S_{1}(ATP_{n})=(1-d{AMP}_{n}/d{ATP}_{n}{)}^{-1}$ and $S_{2}(ATP_{g})=(1-d{AMP}_{g}/d{ATP}_{g}{)}^{-1}$, which are dependent on *ATP*_*n*_ and *ATP*_*g*_, respectively. However, such a formulation complicates the employment of the CSP tools. We thus reformulated the system of equations to match the form of Eq (4), which requires the stoichiometry to be independent of the variables of the model. Each reaction rate included in the governing equations of *ATP*_*n*_ or *ATP*_*g*_ is now reformulated as the product of the corresponding flux in [25] times $S_{1}(ATP_{n})$ or $S_{1}(ATP_{g})$, respectively, as shown in Tables 6.

**Table 4:** Governing equations.

Variable	Value at rest			Differential equation
Intracellular sodium	8/15	mM	$\frac{d}{dt}Na_{x}^{+}=$	$R^{1}-3R^{3}+R^{40}$
				$R^{1}-3R^{4}+R^{41}$
Neuronal glucose	1.2	mM	$\frac{d}{dt}GLC_{n}=$	(*R^5f^*–*R^5b^*)–*R*^9^
Astrocytic glucose	1.19	mM	$\frac{d}{dt}GLC_{g}=$	$(R^{6f}-R^{6b})+(R^{7f}-R^{7b})-R^{10}$
Glyceraldehyde-3-phosphate	0.0046	mM	$\frac{d}{dt}GAP_{x}=$	2*R*^9^–*R*^11^
				2*R*^10^–*R*^12^
Phosphoenolpyruvate	0.015	mM	$\frac{d}{dt}PEP_{x}=$	*R*^11^–*R*^13^
				*R*^12^–*R*^14^
Pyruvate	0.17	mM	$\frac{d}{dt}PYR_{x}=$	*R*^13^–(*R^15f^*–*R^15b^*)–*R*^21^
				*R*^14^–(*R*${\;}^{16\text{f}}$–*R*${\;}^{16\text{b}}$)–*R*^23^
Neuronal lactate	0.6	mM	$\frac{d}{dt}LAC_{n}=$	(*R*${\;}^{15\text{f}}$–*R*${\;}^{15\text{b}}$)–(*R*${\;}^{17\text{f}}$–*R*${\;}^{17\text{b}}$)
Astrocytic lactate	0.6	mM	$\frac{d}{dt}LAC_{g}=$	(*R*${\;}^{16\text{f}}$–*R*${\;}^{16\text{b}}$)–(*R*${\;}^{18\text{f}}$–*R*${\;}^{18\text{b}}$)–(*R*${\;}^{19\text{f}}$–*R*${\;}^{19\text{b}}$)
Cytosolic NADH	0.006/0.1	mM	$\frac{d}{dt}NADH_{x}^{cyto}=$	$c_{1}(R^{11}-(R^{15f}-R^{15b})-R^{25})$
				$c_{1}(R^{12}-(R^{16f}-R^{16b})-R^{26})$
Mitochondrial NADH	0.12	mM	$\frac{d}{dt}NADH_{x}^{mito}=$	$c_{2}(4R^{21}-R^{22}+R^{25})$
				$c_{2}(4R^{23}-R^{24}+R^{26})$
Neuronal ATP	2.2	mM	$\frac{d}{dt}ATP_{n}=$	$-2R^{54}+R^{56}+R^{58}-R^{34}-R^{60}+3.6R^{62}+R^{64f}-R^{64b}$
Astrocytic ATP	2.2	mM	$\frac{d}{dt}ATP_{g}=$	$-2R^{55}+R^{57}+R^{59}-R^{35}+0.75R^{36}-1.75R^{61}+3.6R^{63}+R^{65f}-R^{65b}$
Phosphocreatine	4.9	mM	$\frac{d}{dt}PCr_{x}=$	–(*R*${\;}^{27\text{f}}$–*R*${\;}^{27\text{b}}$)
				–(*R*${\;}^{28\text{f}}$–*R*${\;}^{28\text{b}}$)
Intracellular oxygen	0.028	mM	$\frac{d}{dt}O_{2x}=$	*R*^29^–0.6*R*^22^
				*R*^30^–0.6*R*^24^
Capillary oxygen	7	mM	$\frac{d}{dt}O_{2c}=$	$R^{31}-r_{1}R^{29}-r_{2}R^{30}$
Capillary glucose	4.5	mM	$\frac{d}{dt}GLC_{c}=$	$R^{32}-r_{2}(R^{7f}-R^{7b})-r_{3}(R^{8f}-R^{8b})$
Capillary lactate	0.55	mM	$\frac{d}{dt}LAC_{c}=$	$R^{33}-r_{2}(R^{19f}-R^{19b})+r_{3}(R^{20f}-R^{20b})$
Venous volume	0.02		$\frac{d}{dt}V_{v}=$	*R* ^37^
Deoxyhemoglobin	0.058	mM	$\frac{d}{dt}dHb=$	*R*^38^–*R*^39^
Extracellular glucose	2.48	mM	$\frac{d}{dt}GLC_{e}=$	$-r_{4}(R^{5f}-R^{5b})-r_{5}(R^{6f}-R^{6b})+(R^{8f}-R^{8b})$
Extracellular lactate	0.6	mM	$\frac{d}{dt}LAC_{e}=$	$r_{4}(R^{17f}-R^{17b})+r_{5}(R^{18f}-R^{18b})-(R^{20f}-R^{20b})$

When two values are indicated, the first one corresponds to the neuronal (*n*) compartment and the second one to the astrocytic (glial) compartment (*g*). *x* denotes either *n* or *g*, evolving according to the first and second equation in each row, respectively. NADH stands for nicotinamide adenine dinucleotide and ATP for adenosine triphosphate.

**Table 5:** Reaction and transport rates (except for equations governing the dynamics of ATP).

Reaction or transport	Rate	Expression	In ref. [25]
Sodium leak	*R*^1^,*R*^2^	$=\frac{S_{m}V_{x}}{F}g_{Na}^{x}\left[\frac{RT}{F}\log({Na}_{e}^{+}/{Na}_{x}^{+})-\psi_{x}\right]$	$J_{leak,Na}^{x}$
Na,K-ATPases	*R*^3^,*R*^4^	$=S_{m}V_{x}k_{pump}^{x}{ATP}_{x}{Na}_{x}^{+}(1+\frac{{ATP}_{x}}{K_{m,pump}}{)}^{-1}$	$J_{pump}^{x}$
Glucose transport	*R*${\;}^{5\text{f}}$–*R*${\;}^{5\text{b}}$, *R*${\;}^{6\text{f}}$–*R*${\;}^{6\text{b}}$	$=T_{max,GLC}^{xy}\left(\frac{{GLC}_{x}}{{GLC}_{x}+K_{GLC}^{xy}}-\frac{{GLC}_{y}}{{GLC}_{y}+K_{GLC}^{xy}}\right)$	$J_{GLC}^{xy}$
	*R*${\;}^{7\text{f}}$–*R*${\;}^{7\text{b}}$, *R*${\;}^{8\text{f}}$–*R*${\;}^{8\text{b}}$		
Hexokinase-phosphofructokinase	*R*^9^,*R*^10^	$=k_{HKPFK}^{x}{ATP}_{x}\frac{{GLC}_{x}}{{GLC}_{x}+K_{g}}{\left[1+(\frac{{ATP}_{x}}{K_{I,ATP}}{)}^{nH}\right]}^{-1}$	$J_{HKPFK}^{x}$
Phosphoglycerate kinase	*R*^11^,*R*^12^	$=k_{PGK}^{x}{GAP}_{x}{ADP}_{x}\;\frac{N-{NADH}_{x}^{cyto}}{{NADH}_{x}^{cyto}}$	$J_{PGK}^{x}$
Pyruvate kinase	$R^{13},R^{14}$	$=k_{PK}^{x}{PEP}_{x}{ADP}_{x}$	$J_{PK}^{x}$
Lactate dehydrogenase	*R*${\;}^{15\text{f}}$–*R*${\;}^{15\text{b}}$	$=k_{LDH}^{x+}{PYR}_{x}{NADH}_{x}^{cyto}-k_{LDH}^{x-}{PYR}_{x}\left(N-{NADH}_{x}^{cyto}\right)$	$J_{LDH}^{x}$
	*R*${\;}^{16\text{f}}$–*R*${\;}^{16\text{b}}$		
Lactate transport	*R*${\;}^{17\text{f}}$–*R*${\;}^{17\text{b}}$,*R*${\;}^{18\text{f}}$–*R*${\;}^{18\text{b}}$	$=T_{max,LAC}^{xy}\left(\frac{{LAC}_{x}}{{LAC}_{x}+K_{LAC}^{xy}}-\frac{{LAC}_{y}}{{LAC}_{y}+K_{LAC}^{xy}}\right)$	$J_{LAC}^{xy}$
	*R*${\;}^{19\text{f}}$–*R*${\;}^{19\text{b}}$,*R*${\;}^{20\text{f}}$–*R*${\;}^{20\text{b}}$		
TCA cycle	*R*^21^,*R*^23^	$=V_{max,in}^{x}\frac{{PYR}_{x}}{{PYR}_{x}+K_{m}^{mito}}\;\frac{N-{NADH}_{x}^{mito}}{N-{NADH}_{x}^{mito}+K_{m,NAD}^{x}}$	$J_{mito,in}^{x}$
Electron transport chain	*R*^22^,*R*^24^	$=V_{max,out}^{x}\frac{O_{2x}}{O_{2x}+K_{O_{2}}^{mito}}\;\frac{{ADP}_{x}}{{ADP}_{x}+K_{m,ADP}^{x}}\;\frac{{NADH}_{x}^{mito}}{{NADH}_{x}^{mito}+K_{m,NADH}^{x}}$	$J_{mito,out}^{x}$
NADH shuttles	*R*^25^,*R*^26^	$=T_{NADH}^{x}\frac{R_{x}^{-}}{R_{x}^{-}+M_{x}^{cyto}}\;\frac{R_{x}^{+}}{R_{x}^{+}+M_{x}^{mito}}$	$J_{shuttle}^{x}$
Creatine kinase	*R*${\;}^{27\text{f}}$–*R*${\;}^{27\text{b}}$	$=k_{CK}^{x+}{ADP}_{x}{PCr}_{x}-k_{CK}^{x-}{ATP}_{x}\left(C-{PCr}_{x}\right)$	$J_{CK}^{x}$
	*R*${\;}^{28\text{f}}$–*R*${\;}^{28\text{b}}$		
Oxygen exchange	*R*^29^,*R*^30^	$=\frac{PS_{cap}}{V_{x}}\left(K_{O_{2}}(\frac{Hb.OP}{O_{2c}}-1{)}^{-1/nh}-O_{2n}\right)$	$J_{O_{2}m}^{cx}$
Capillary oxygen flow	*R* ^31^	$=\frac{2F_{0}}{V_{cap}}\left(O_{2a}-O_{2c}\right)$	$J_{O_{2}}^{c}$
Capillary glucose flow	*R* ^32^	$=\frac{2F_{0}}{V_{cap}}\left(GLC_{a}-GLC_{c}\right)$	$J_{GLC}^{c}$
Capillary lactate flow	*R* ^33^	$=\frac{2F_{0}}{V_{cap}}\left(LAC_{a}-LAC_{c}\right)$	$J_{LAC}^{c}$
Venous volume	*R* ^37^	$=F_{0}-F_{out}$	
Deoxyhemoglobin flow	*R*^38^–*R*^39^	$=F_{0}\left(O_{2a}-O_{2c}\right)-F_{out}\frac{dHb}{V_{v}}$	

When two reaction rates are indicated, the first corresponds to the neuronal compartment, while the second to the glial one (the notation *x* in the expression is either *n* or *g*). The 2 quadruplet forward-backward pairs of transport rates with the notation *xy* denote transport of the respective species from the *x* compartment to the *y* compartment. ADP is given by Eq (1). $R_{x}^{-}={NADH}_{x}^{cyto}/\left(N-{NADH}_{x}^{cyto}\right)$ and $R_{x}^{+}={(N-NADH}_{x}^{mito})/{NADH}_{x}^{mito}$. The flow out of the venous balloon is given by $F_{out}=F_{0}\left[(\frac{V_{v}}{V_{v0}}{)}^{1/\alpha_{v}}+{\frac{\tau_{v}}{V_{v0}}\left(\frac{V_{v}}{V_{v0}}\right)}^{-1/2}\frac{dV_{v}}{dt}\right]$.

**Table 6:** Reaction rates governing the dynamics of ATP.

Reaction	Rate	Expression	In Ref [25]
ATP offset	$R^{34},R^{35}$	$S_{1}J_{ATPases\;}^{n},S_{2}J_{ATPases}^{g}$	$S_{1}J_{ATPases\;}^{n},S_{2}J_{ATPases}^{g}$
Astrocytic Na,K-ATPase offset	*R* ^36^	$S_{2}J_{pump,0}^{g}$	$S_{2}J_{pump,0}^{g}$
Hexokinase-phosphofructokinase	$R^{54},R^{55}$	$S_{1}R^{9},S_{2}R^{10}$	$S_{1}J_{HKPFK\;}^{n},S_{2}J_{HKPFK}^{g}$
Phosphoglycerate kinase	$R^{56},R^{57}$	$S_{1}R^{11},S_{2}R^{12}$	$S_{1}J_{PGK\;}^{n},S_{2}J_{PGK}^{g}$
Pyruvate kinase	$R^{58},R^{59}$	$S_{1}R^{13},S_{2}R^{14}$	$S_{1}J_{PK\;}^{n},S_{2}J_{PK}^{g}$
Na,K-ATPases	$R^{60},R^{61}$	$S_{1}R^{3},S_{2}R^{4}$	$S_{1}J_{pump\;}^{n},S_{2}J_{pump}^{g}$
Electron transport chain	$R^{62},R^{63}$	$S_{1}R^{22},S_{2}R^{24}$	$S_{1}J_{mito,out\;}^{n},S_{2}J_{mito,out}^{g}$
Creatine kinase	*R*${\;}^{64\text{f}}$,*R*${\;}^{65\text{f}}$	$S_{1}R^{27f},S_{2}R^{28f}$	$S_{1}J_{CK\;}^{n},S_{2}J_{CK}^{g}$
	*R*${\;}^{64\text{b}}$,*R*${\;}^{65\text{b}}$	$S_{1}R^{27b},S_{2}R^{28b}$	

The multiplying factors *S*_1_ and *S*_2_ are *ATP*_*n*_- and *ATP*_*g*_-dependent (neglected in the table for simplicity) since, $S_{1}(ATP_{n})=(1-d{AMP}_{n}/d{ATP}_{n}{)}^{-1}$ and $S_{2}(ATP_{g})=(1-d{AMP}_{g}/d{ATP}_{g}{)}^{-1}$, as shown by the ratio given in Eq 2.

All rates and state variables included in Tables 4, 5 and 6 are given per unit cell volume (neuron or astrocyte), or per unit capillary volume, with the exception of $J_{GLC}^{ce}$, $J_{LAC}^{ec}$, *LAC*_*e*_ and *GLC*_*e*_, which are given per unit extracellular volume. Mitochondrial and cytosolic NADH levels are given per unit mitochondrial or cytosolic volume, respectively. All parameters are given in Table 7.

**Table 7:** Parameters of the model (from Ref [25]).

**Volume fractions:**	$V_{e}=0.2$, $V_{cap}=0.0055$, $V_{g}=0.25$, $V_{n}=0.45$, ξ=0.07 (mitochondrial volume fraction $=V_{mito}/V_{cell}$), $r_{en}=V_{e}/V_{n}$, $r_{eg}=V_{e}/V_{g}$, $r_{ce}=V_{cap}/V_{e}$, $r_{cn}=V_{cap}/V_{n}$, $r_{cg}=V_{cap}/V_{g}$
**Surface to volume ratios:**	$S_{m}V_{n}=2.5\;10^{4}\;cm^{-1}$, $S_{m}V_{g}=2.5\;10^{4}\;cm^{-1}$
**Physical constants:**	$R=8.31451\;Jmol^{-1}K^{-1}$, $F=9.64853\;10^{4}\;Cmol^{-1}$, RT/F=26.73 mV (corresponds to body temperature), $\psi_{n}=-73.59\;mV$, $\psi_{g}=-70\;mV$, $Na_{e}^{+}=150\;mM$
**Na,K-ATPase and sodium**	$g_{Na}^{n}=0.0136$, $g_{Na}^{g}=0.0061$, $g_{K}^{pas}=0.2035\;mScm^{-2}$, $k_{pump}^{n}=2.2\;10^{-6}$, $k_{pump}^{g}=4.5\;10^{-7}\;cmmM^{-1}s^{-1}$,
**leak:**	$J_{pump,0}^{g}=0.0687\;mMs^{-1}$, $K_{m,pump}=0.5\;mM$
**Glucose exchanges**	$T_{max,GLC}^{en}=0.041$, $T_{max,GLC}^{ce}=0.239$, $T_{max,GLC}^{eg}=0.147$, $T_{max,GLC}^{cg}=0.0016\;mMs^{-1}$
**constants and affinities:**	$K_{t,GLC}^{en}=8$, $K_{t,GLC}^{eg}=8$, $K_{t,GLC}^{cg}=8$, $K_{t,GLC}^{ge}=8\;mM$
Hexokinase- phosphofructokinase:	$k_{HKPFK}^{n}=0.0504$, $k_{HKPFK}^{g}=0.185\;s^{-1}$, $K_{I,ATP}=1\;mM$, *nH* = 4, $K_{g}=0.05\;mM$
**Phosphoglycerate kinase:**	$k_{PGK}^{n}=3.97$, $k_{PGK}^{g}=135.2\;mM^{-1}s^{-1}$
**Pyruvate kinase:**	$k_{PK}^{n}=36.7$, $k_{PK}^{g}=401.7\;mM^{-1}s^{-1}$
**Lactate dehydrogenase:**	$k_{LDH}^{n+}=72.3$, $k_{LDH}^{g+}=1.59\;Lmmol^{-1}s^{-1}$, $k_{LDH}^{n-}=0.72$, $k_{LDH}^{g-}=0.071\;Lmmol^{-1}s^{-1}$
**Lactate exchanges**	$T_{max,LAC}^{gc}=0.00243$, $T_{max,LAC}^{ne}=24.3$, $T_{max,LAC}^{ge}=106.1$, $T_{max,LAC}^{ec}=0.25\;mMs^{-1}$
constants and affinities:	$K_{t,LAC}^{en}=0.74$, $K_{t,LAC}^{ge}=3.5$, $K_{t,LAC}^{gc}=1.0$, $K_{t,LAC}^{ec}=1.0\;mM$
**TCA cycle:**	$V_{max,in}^{n}=0.1303$, $V_{max,in}^{g}=5.7\;mMs^{-1}$, $K_{m}^{mito}=0.04\;mM$, $K_{m,NAD}^{n}=0.409$, $K_{m,NAD}^{g}=40.3\;mM$
**Electron transport chain:**	$K_{m,ADP}^{n}=3.41\;10^{-3}$, $K_{m,ADP}^{g}=0.483\;10^{-3}$, $K_{m,NADH}^{n}=4.44\;10^{-2}$, $K_{m,NADH}^{g}=2.69\;10^{-2}\;mM$, $K_{O_{2}}^{mito}=0.001\;mM$, $V_{max,out}^{n}=0.164$, $V_{max,out}^{g}=0.064\;mMs^{-1}$
**NADH shuttles:**	$M_{n}^{cyto}=4.9\;10^{-8}$, $M_{g}^{cyto}=2.5\;10^{-4}$, $M_{n}^{mito}=3.93\;10^{5}$, $M_{g}^{mito}=1.06\;10^{4}$ $T_{NADH}^{n}=10330$, $T_{NADH}^{g}=150\;mMs^{-1}$
**Total creatine plus phospho- creatine concentration**	C=10 mM
**Creatine kinase:**	$k_{CK}^{n+}=0.0433$, $k_{CK}^{g+}=0.00135\;mM^{-1}s^{-1}$, $k_{CK}^{n-}=0.00028$, $k_{CK}^{g-}=10^{-5}\;mM^{-1}s^{-1}$
**Oxygen exchange constants:**	$K_{O_{2}}=0.0361\;mM$, Hb.OP=8.6 mM, *nh* = 2.73, $PS_{cap}/V_{n}=1.66$, $PS_{cap}/V_{g}=0.87\;s^{-1}$
**Blood flow contribution to capillary oxygen glucose and lactate**	*O*_2*a*_ = 8.35, $GLC_{a}=4.75\;mM$, $LAC_{a}=0.506\;mM$
**Venous balloon:**	$\tau_{\nu }=35\;s$, $\alpha_{\nu }=0.5$
**Total nicotinamide adenine dinucleotide concentration**	N=212 mM
**ATPases:**	$J_{ATPases}^{n}=0.1695$, $J_{ATPases}^{g}=0.1404\;mMs^{-1}$

Additionally, the model includes the following equations as a result of energy conservation within neurons and astrocytes. *ADP*_*x*_ is given as a function of the ATP concentration (*x* stands for *n* or *g*) as:

$$ADP_{x}=\frac{ATP_{x}}{2}\left[-q_{AK}+\sqrt{q_{AK}^{2}+4q_{AK}\left(A/ATP_{x}-1\right)}\right],$$ (1)

with $A=AMP_{x}+ADP_{x}+ATP_{x}=2.212\;$mM the total adenine nucleotide concentration and *q*_*AK*_ = 0.92, the adenylate kinase equilibrium constant. As a consequence:

$$\frac{dAMP_{x}}{dATP_{x}}=-1+\frac{q_{AK}}{2}-\frac{1}{2}\sqrt{u_{x}}+\frac{q_{AK^{A}}}{ATP_{x}\sqrt{u_{x}}},$$ (2)

with $u_{x}=q_{AK}^{2}+4q_{AK}\left(A/ATP_{x}-1\right)$.

The model receives two separate inputs during activation, due to modulation of (i) the presynaptic excitatory population, and (ii) the cerebral blood flow, modulated by neurovascular coupling.

The first input from the presynaptic excitatory population is a result of glutamate release during activation. In particular, the glutamate released by excitatory presynaptic neurons drives the intracellular sodium concentration in both the neuronal and glial compartments, and is the main driver of downstream energy metabolism. This is formulated in the model in reactions 40 and 41, which take the constant values $R^{40}=v_{stim}^{n}=1.5290\;10^{-1}$ and $R^{41}=v_{stim}^{g}=5.9823\;10^{-2}$ during activation, and zero afterwards. Details about how these reactions are calculated can be found in [25].

The second input relates to the cerebral blood flow, which upon activation is modelled as a trapezoidal function. It linearly rises over 30 s with a time lag of 2 s at the onset of activation, and of 10 s at the offset of activation, as implemented for the human brain scenario in [25]; the baseline value is $F_{0}=0.012\;s^{-1}$, while the one attained during activation is $1.4\cdot F_{0}=0.0168\;s^{-1}$. The Blood-Oxygen-Level-Dependent (BOLD) signal can then be computed as a function of the deoxyhemoglobin concentration (*dHb*) and of the venous volume ($V_{v}$):

$$BOLD(t)=V_{V,0}\left[\left(k_{1}+k_{2}\right)\left(1-\frac{dHb}{dHb_{0}}\right)-\left(k_{2}+k_{3}\right)\left(1-\frac{V_{V}}{V_{V,0}}\right)\right],$$ (3)

with dimensionless parameters *k*_1_ = 2.22, *k*_2_ = 0.46 and *k*_3_ = 0.43 [27]. The steady-state values of deoxyhemoglobin (*dHb*_0_) and venous volume ($V_{V,0}$) are given in Table 4.

### Computational Singular Perturbation (CSP) method and algorithmic tools

The mathematical model described above can be formulated as a system of Ordinary Differential Equations (ODEs) in the form:

$$\frac{d𝐲}{dt}=\sum_{k=1}^{K}{𝐒}_{k}R^{k}(𝐲)=g(y),$$ (4)

where y is the *N*-dimensional column vector, the elements of which are the chemical species’ concentrations, $S_{k}$ denotes the *N*-dimensional stoichiometric vector of the *K* unidirectional reactions or currents, and *R*_*k*_ is the related reaction or current rate. In the analysis that follows, all the reversible reactions’ contributions $S_{k}R^{k}$ will be considered separately as two unidirectional reactions, $S_{k}R^{k,f}(𝐲)$ and $S_{k}R^{k,b}(𝐲)$, respectively. The system in Eq (4) can be cast in the following form [30,62]:

$$\frac{d𝐲}{dt}=𝐠(𝐲)=\sum_{n=1}^{N}{𝐚}_{n}(𝐲)f^{n}(𝐲),\;f^{n}(𝐲)={𝐛}^{n}(𝐲)\cdot g(y)=\sum_{k=1}^{K}\left({𝐛}^{n}(𝐲)\cdot {𝐒}_{k}\right)R^{k}(𝐲),$$ (5)

where ${𝐚}_{n}(𝐲)$ and ${𝐛}^{n}(𝐲)$ are the *N*-dim. CSP column and row, respectively, basis vectors of the *n*-th mode, which satisfy the orthogonality conditions ${𝐛}^{i}(𝐲)$ ⋅ ${𝐚}_{j}(𝐲)=\delta_{j}^{i}$ [30,63]. $f^{n}(𝐲)$ is the amplitude of the *n*-th mode (proper adjustment of the signs of the CSP basis vectors set $f^{n}(𝐲)$ always positive), which provides a measure of the projection of the vector field g(y) on the CSP vector ${𝐚}_{n}$. When the system in Eq 5 exhibits *M* timescales that are [i] of dissipative nature (i.e., when the components of the system that generate them tend to drive the system towards equilibrium) and [ii] much faster than the rest, the model can be reduced in:

$${f\;}^{r}(𝐲)\approx 0\;(r=1,\ldots ,M),\;\frac{d𝐲}{dt}\approx \sum_{s=M+1}^{N}{𝐚}_{s}(𝐲)f^{s}(𝐲),$$ (6)

the first relation of which is an *M*-dim. system of algebraic equations defining the manifold M (a low dimensional surface in phase-space, where the system is confined to evolve), while the second relation is an *N*-dim. system of ODEs governing the slow evolution of the system on this manifold. By providing the two expressions in Eq 6, CSP can generate, order by order, the results of asymptotic analysis [64,65]. The CSP vectors ${𝐚}_{i}(𝐲)$ and ${𝐛}^{i}(𝐲)$ (i=1,…,N) can be approximated in leading order accuracy by the right and left, respectively, eigenvectors of the *N* × *N*-dim. Jacobian J(y) of g(y); i.e., ${𝐚}_{i}(𝐲)=\alpha_{i}(𝐲)$ and ${𝐛}^{i}(𝐲)=\beta^{i}(𝐲)$ [30,62,66]. In the following, the dependency of *R*^*k*^, **g**, α, etc... from **y** will be removed for simplicity.

The equilibria among the reactions that are established by the fast dynamics are expressed by the *M* constraints in Eq 6 ${f\;}^{r}=(\beta^{r}\cdot {𝐒}_{1})R^{1}+\ldots +(\beta^{r}\cdot {𝐒}_{K})R^{K}\approx 0$ (r=1,…,M), as a result of significant cancellations among some of the additive terms $(\beta^{r}\cdot {𝐒}_{k})R^{k}$ (k=1,…,K). The reactions that contribute significantly to the formation of each of the *M* constraints are identified by the *Amplitude Participation Index* (*API*):

$$P_{k}^{r}=\frac{(\beta^{r}{𝐒}_{k})R^{k}}{\sum_{i=1}^{K}|(\beta^{n}{𝐒}_{i})R^{i}|}\;(k=1,\ldots ,K),$$ (7)

where by definition $\sum_{k=1}^{K}|P_{k}^{r}|=1$ [32,33,63]. The relative contribution of the *k*-th reaction to the cancellations among the additive terms in ${f\;}^{r}\approx 0$ is thus measured by $P_{k}^{r}$, which can be either positive or negative; the sum of positive and negative terms equaling 0.5, by definition.

The formation of the *M* constraints is characterized by the *M* fastest timescales, while the dynamics of the slow system in Eq 6 by the fastest of the *N*–*M* slow ones. The timescales are approximated by the inverse of the eigenvalues of the Jacobian **J**, $\tau_{n}=|\lambda_{n}{|}^{-1}$ (n=1,…,N). The CSP diagnostic tool, *Timescale Participation Index* (*TPI*), identifies the reactions significantly contributing to the generation of the timescales:

$$J_{k}^{n}=\frac{c_{k}^{n}}{\sum_{i=1}^{K}|c_{i}^{n}|}\;(k=1,\ldots ,K),$$ (8)

where $\lambda_{n}=c_{1}^{n}+\ldots +c_{K}^{n}$ and by definition $\sum_{k=1}^{K}|J_{k}^{n}|=1$ [33,67,68]. $c_{k}^{n}$ denotes the contribution of the *k*-th reaction to the *n*-th eigenvalue and can be calculated as $c_{k}^{n}=\beta^{n}\nabla \left({𝐒}_{k}R^{k}\right)\alpha_{n}$, where $\sum_{k=1}^{K}\nabla \left({𝐒}_{k}R^{k}\right)$ is the Jacobian **J**. $c_{k}^{n}$ can be either positive or negative and therefore, a negative (positive) $J_{k}^{n}$ implies that the *k*-th reaction contributes to a dissipative (explosive) character of the *n*-th timescale $\tau_{n}$. By definition, dissipative (explosive) timescales relate to the components of the system that tend to drive it towards (away from) equilibrium [30,63].

Each CSP mode is associated differently to each metabolic species. The relation of the *m*-th CSP mode (m=1,…,M) to the various chemical species is identified by the *Pointer* (*Po*):

$${𝐃}^{m}=diag\left[\alpha_{m}\beta^{m}\right]=\left[\alpha_{m}^{1}\beta_{1}^{m},\alpha_{m}^{2}\beta_{2}^{m},\ldots ,\alpha_{m}^{N}\beta_{N}^{m}\right]\;(m=1,\ldots ,M),$$ (9)

where, due to the orthogonality condition $\beta^{i}\cdot \alpha_{j}=\delta_{j}^{i}$, the sum of all *N* elements of ${𝐃}^{m}$ equals unity, i.e. $\sum_{i=1}^{N}\alpha_{m}^{i}\beta_{i}^{m}=1$ [32,39,63,69]. A relatively large value of $\alpha_{m}^{i}\beta_{i}^{m}$ indicates that the *i*-th species is strongly associated to *m*-th CSP mode and the *m*-th timescale. A value of $D_{i}^{m}$ close to unity suggests that the *i*-th variable is in *Quasi Steady-State* (QSS) [39].

The identification of the number *M* of exhausted modes is algorithmically provided by CSP using the criterion:

$$\left|\tau_{M+1}\sum_{i=1}^{M}\alpha_{i}f^{i}\right|<e_{rel}y+e_{abs},$$ (10)

where *e*_*rel*_ and $e_{abs}$ denote relative and absolute error, respectively. Given the solution of the system at a specific time point, the criterion in Eq 10 identifies the *M* fast timescales, according to the desired accuracy. When *M* timescales are considered exhausted, the magnitude of the (*M*  +  1)-th CSP amplitude, *f*^*M* + 1^, is among the amplitudes with the strongest impact (see Fig D in S1 Text for the (*M* + 1)-th CSP amplitude during each period).

### Applying CSP to the model of brain energy metabolism

The step-by-step workflow can be broken down into the following steps. First, we performed a numerical simulation of the complete biophysical model as described above. This step generated the raw time-course data for all metabolite concentrations and reaction rates.

The core CSP analysis was then applied as a post-processing step to the simulation output. At each time step, we computed the system’s Jacobian matrix. The CSP algorithm then used this matrix to calculate the complete set of timescales of the system, and to classify the corresponding modes at each time point as either fast or slow. The process of identifying timescales and decomposing the dynamics of the system is agnostic to the specific biological context, network topology, or to the number of governing equations. As long as a system can be described by ODEs, the CSP algorithm can be applied to it. The scalability of this approach is thus only limited by practical computational resources.

This initial analysis provides a time series that captures the evolution of the system’s timescales over time (as visualized in Fig 2). We then algorithmically processed this output to identify distinct consecutive periods (*P*_1_ to *P*_8_). A period is formally defined as a time interval during which the number of fast, exhausted modes remains constant. The transition from one period to the next signifies a functional shift in the underlying dynamics of the system.

Once these periods were defined, we chose a representative time point within each (see Tables 1, 2 and 3) to carry out a more detailed investigation using a suite of CSP diagnostic tools. The key tools used were: Amplitude Participation Index (API) and Pointer (Po), used to identify the specific reactions and metabolites that significantly contribute to the fast, equilibrated dynamics within that period; and Timescale Participation Index (TPI) and CSP Pointer (Po), used to identify the key reactions and metabolites that govern the dominant slow dynamics that are driving the system’s evolution during that period.

The quantitative output of these tools is what populates Tables 1, 2 and 3 and informs the schematic diagrams in Figs 3 and 4, which we then interpreted to draw biological conclusions.

## Supporting information

S1 Text Supplementary text. (PDF)
