# Supplementary material for: Elucidating reaction dynamics in a model of human brain energy metabolism
Source: PLoS Comput Biol. 2025 Sep 24;21(9):e1013504. doi: 10.1371/journal.pcbi.1013504 (PMC12500149; doi:10.1371/journal.pcbi.1013504)
Supplement: S1 Text — (PDF) [file pcbi.1013504.s001.pdf]

Elucidating Reaction Dynamics in a Model of Human Brain Energy Metabolism

Dimitris G. Patsatzis[1*], Efstathios-Al. Tingas[2], S. Mani Sarathy[3], Dimitris A. Goussis[4], Renaud Blaise Jolivet[5*]

1 Scuola Superiore Meridionale (SSM), Modelling Engineering Risk & Complexity (MERC), Napoli, Italy
2 School of Computing, Engineering and the Built Environment, Edinburgh Napier University, Edinburgh EH10 5DT, United Kingdom
3 King Abdullah University of Science and Technology (KAUST), Clean Combustion Research Center (CCRC), Thuwal, Saudi Arabia
4 Department of Mechanical Engineering, Khalifa University of Science, Technology and Research (KUSTAR), Abu Dhabi, United Arab Emirates
5 Maastricht Centre for Systems Biology (MaCSBio), Maastricht University, Maastricht, The Netherlands

* d.patsatzis@ssmeridionale.it (DGP); r.jolivet@maastrichtuniversity.nl (RBJ)

Supplementary Figures

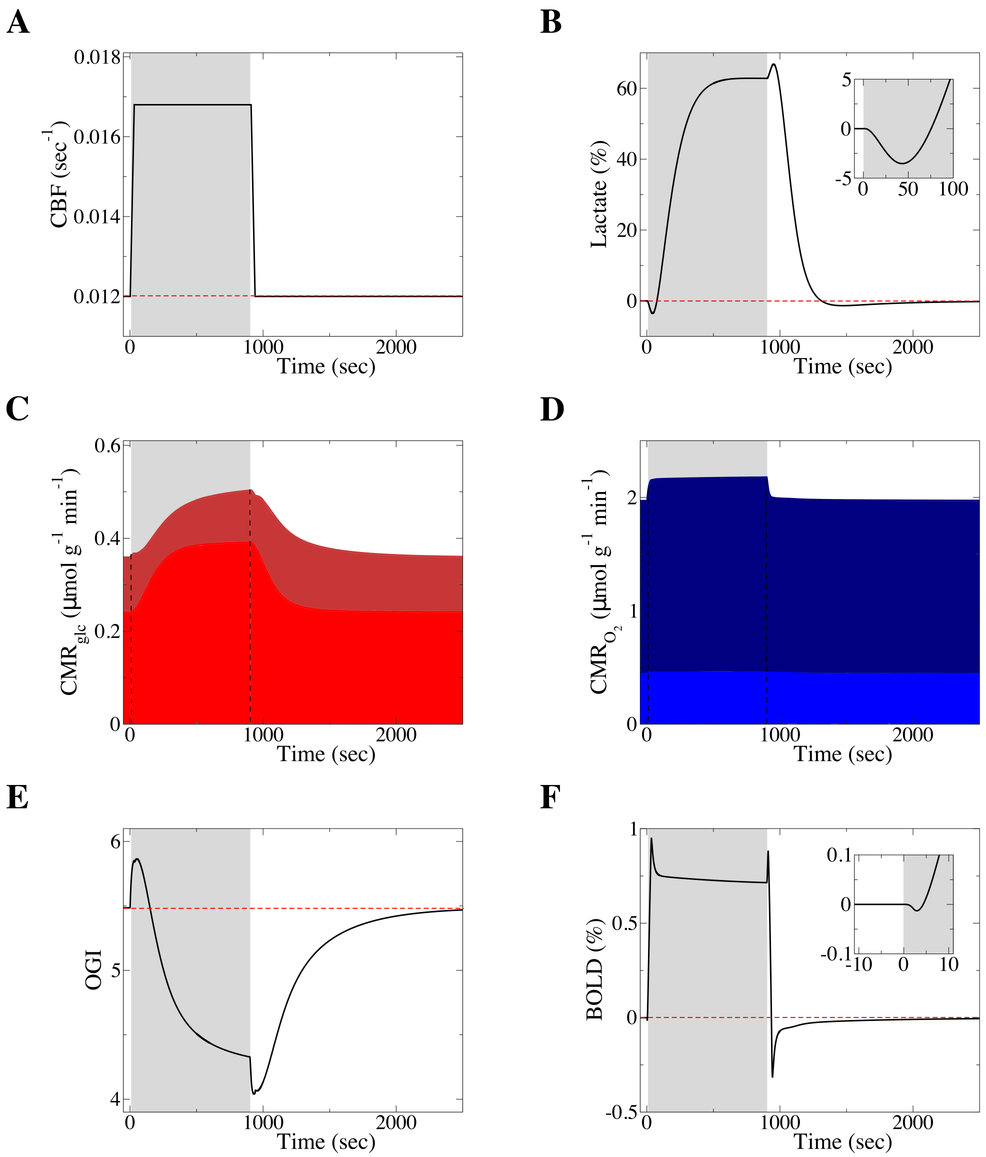

Fig A. Reproduction of the original results by Jolivet and colleagues [1] in the *in vivo* human brain metabolic activation scenario.
(A) Temporal evolution of the cerebral blood flow chosen as an input to the model during a simulated 900 sec stimulation episode *in vivo* (grey area). This specific time course closely matches *in vivo* measurements in humans during imaging experiments [2, 3]. (B) Relative fluctuations of tissue lactate concentration during the same stimulation episode as in A. The model predicts an initial lactate dip followed by a 60% increase sustained till the end of the stimulation. The presence of a dip matches experimental data from Mangia *et al.* [4]. (C and D) Cerebral metabolic rate of glucose consumption (CMR_{glc}) and cerebral metabolic rate of oxygen consumption ($CMRO_2$) during the same 900 sec stimulation episode as in A to B. In both cases, the light area corresponds to the contribution of the astrocytic compartment towards the total tissue consumption, while the dark area corresponds to the contribution of the neuronal compartment. While glucose consumption increases by about 40%, the increase is mostly due to the astrocytic compartment (light red) with the neuronal glucose utilization even slightly decreasing at the onset of activation (dark red). On the contrary, while oxygen utilization increases by about 10%, most of this increase is due to the neuronal compartment (dark blue) with the astrocytic oxygen utilization being almost constant (light blue). (E) The predicted ratio of ($CMRO_2$) to (CMR_{glc}) or oxygen-glucose index (OGI) during the same 900 sec stimulation episode as in A to D. (F) Predicted BOLD signal for the same 900 sec stimulation episode as in A to E. Like the tissue lactate concentration in B, the BOLD shows a clear dip at the onset of activation.

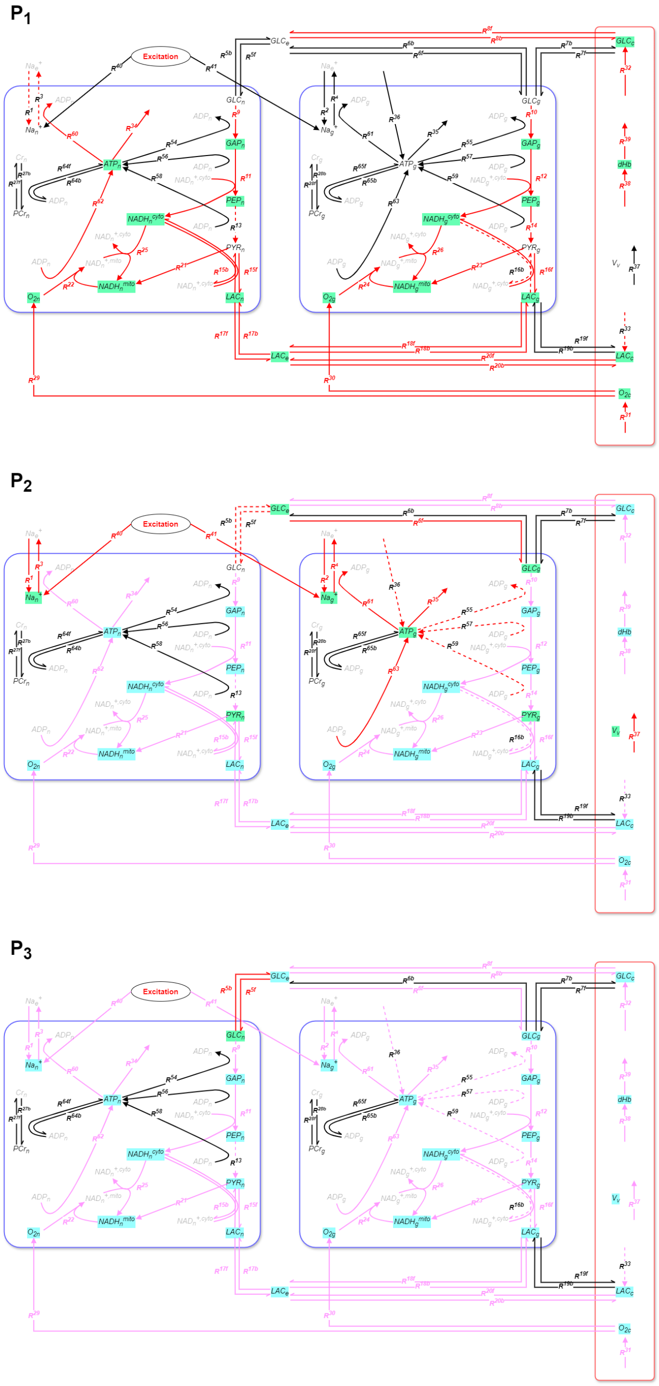

Fig B. Equilibrating reactions and fast metabolic species in periods 1 to 3 (activation epoch). (Top) to (Bottom) Periods P_1 to P_3. In each period, an equilibrium is formed between multiple reactions, and some metabolic species are identified to be related with those fast modes. Reactions that have been identified by the CSP API ($Amplitude\ Participation\ Index$; see Eq. (7)) tool to contribute to the equilibrium formed for each mode are displayed in red (solid lines: contributions $> 9\%$; dashed lines: contributions from 2% to 9%). These reactions then appear in magenta in subsequent periods. Metabolic species highlighted in green are those identified by the CSP Po ($Pointer$; see Eq. (9)) to be more than 45% related to the fast modes. These species then appear in cyan in subsequent periods. Note that the presynaptic excitatory activity (reactions 40 and 41), contributes to the equilibrium formed in periods P_2 and P_3, but not to the equilibrium formed in period P_1.

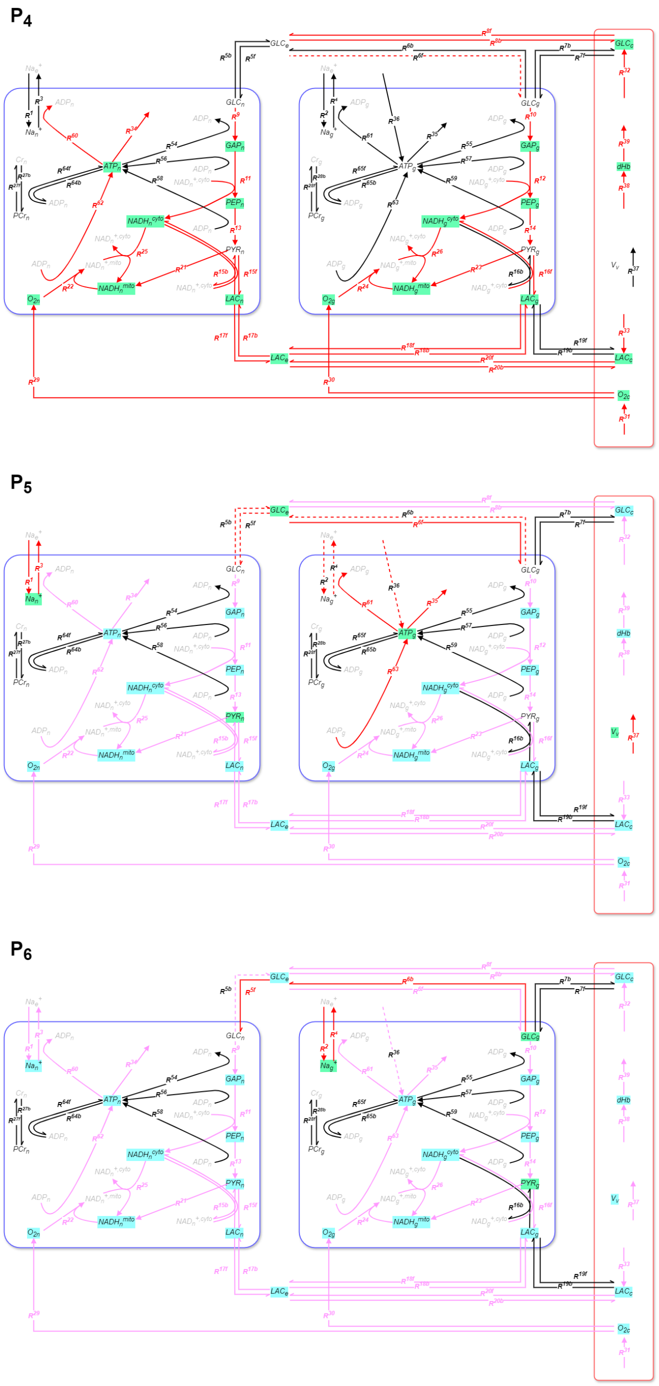

Fig C. Equilibrating reactions and fast metabolic species in periods 4 to 6 (post-activation epoch).

(Top) to (Bottom) Periods P_4 to P_6. In each period, an equilibrium is formed between multiple reactions, and some metabolic species are identified to be related with those fast modes. Reactions that have been identified by the CSP API tool to contribute to the equilibrium formed for each mode are displayed in red (solid lines: contributions $> 9\%$; dashed lines: contributions from 2% to 9%). These reactions then appear in magenta in subsequent periods. Metabolic species highlighted in green are those identified by the CSP Po to be more than 45% related to the fast modes. These species then appear in cyan in subsequent periods.

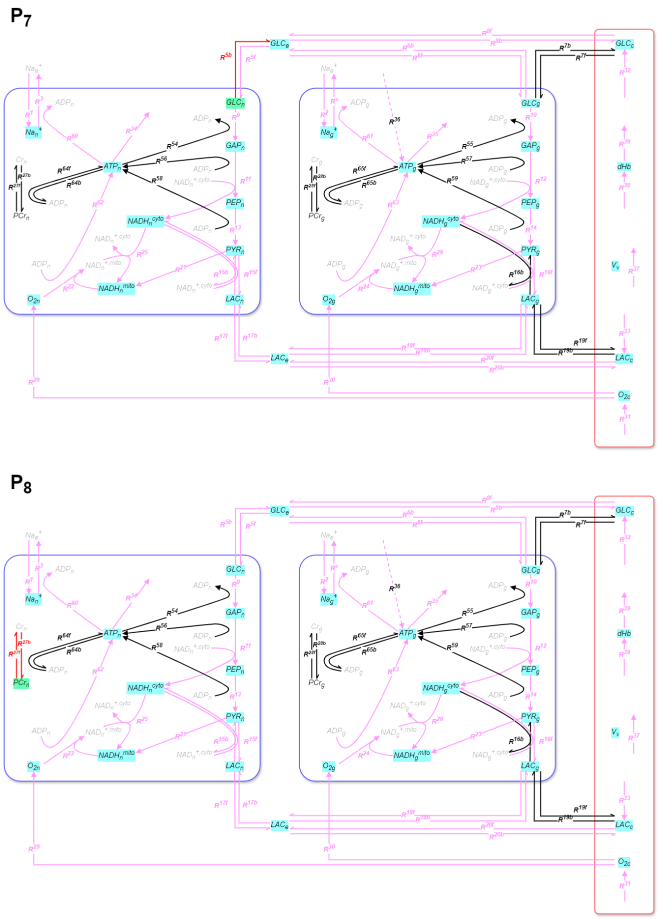

Fig D. Equilibrating reactions and fast metabolic species in periods 7 and 8 (post-activation epoch).
(Top) to (Bottom) Periods P_7 and P_8. In each period, an equilibrium is formed between multiple reactions, and some metabolic species are identified to be related with those fast modes. Reactions that have been identified by the CSP API tool to contribute to the equilibrium formed for each mode are displayed in red (solid lines: contributions $> 9\%$; dashed lines: contributions from 2% to 9%). These reactions then appear in magenta in subsequent periods. Metabolic species highlighted in green are those identified by the CSP Po to be more than 45% related to the fast modes. These species then appear in cyan in subsequent periods.

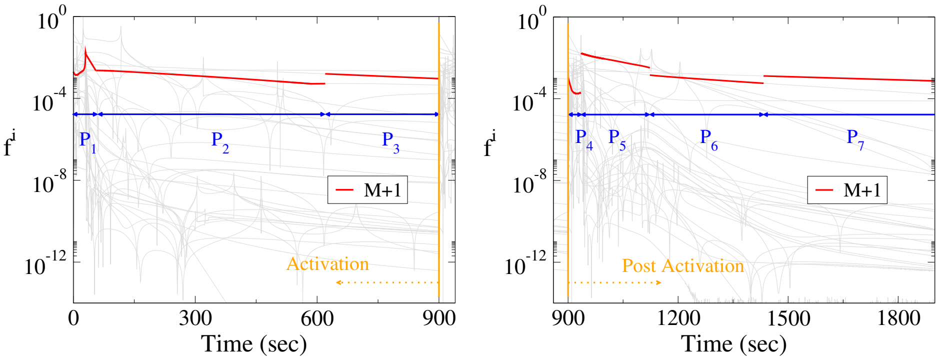

Fig E. Amplitude of the dominant slow mode during the activation and post-activations epochs. The amplitudes f^i, as computed on the basis of the eigenvectors in Eq (5), are shown in grey. The dominant slow mode f^{M+1} in periods P_1-P_3 of the activation epoch (left), and in periods P_4-P_7 of the post-activation epoch, is highlighted in red.

References

1. Jolivet R, Coggan JS, Allaman I, Magistretti PJ. Multi-timescale modeling of activity-dependent metabolic coupling in the neuron-glia-vasculature ensemble. PLOS Computational Biology. 2015;11(2):e1004036.

2. Nakai T, Matsuo K, Kato C, Takehara Y, Isoda H, Moriya T, et al. Post-stimulus response in hemodynamics observed by functional magnetic resonance imaging—Difference between the primary sensorimotor area and the supplementary motor area. Magnetic Resonance Imaging. 2000;18(10):1215–1219.

3. Obata T, Liu TT, Miller KL, Luh WM, Wong EC, Frank LR, et al. Discrepancies between BOLD and flow dynamics in primary and supplementary motor areas: application of the balloon model to the interpretation of BOLD transients. NeuroImage. 2004;21(1):144–153.

4. Mangia S, Garreffa G, Bianciardi M, Giove F, Di Salle F, Maraviglia B. The aerobic brain: lactate decrease at the onset of neural activity. Neuroscience. 2003;118(1):7–10.
